# Mechanisms of Hydride Nucleation, Growth, Reorientation, and Embrittlement in Zirconium: A Review

**DOI:** 10.3390/ma16062419

**Published:** 2023-03-17

**Authors:** Yu-Jie Jia, Wei-Zhong Han

**Affiliations:** Center for Advancing Materials Performance from the Nanoscale, State Key Laboratory for Mechanical Behavior of Materials, Xi’an Jiaotong University, Xi’an 710049, China

**Keywords:** zirconium hydride, nucleation, growth, reorientation, embrittlement

## Abstract

Zirconium (Zr) hydrides threaten the reliability of fuel assembly and have repeatedly induced failures in cladding tubes and pressure vessels. Thus, they attract a broad range of research interests. For example, delayed hydride cracking induced a severe fracture and failure in a Zircaloy-2 pressure tube in 1983, causing the emergency shutdown of the Pickering nuclear reactor. Hydride has high hardness and very low toughness, and it tends to aggregate toward cooler or tensile regions, which initiates localized hydride precipitation and results in delayed hydride cracking. Notably, hydride reorientation under tensile stress substantially decreases the fracture toughness and increases the ductile-to-brittle transition temperature of Zr alloys, which reduces the safety of the long-term storage of spent nuclear fuel. Therefore, improving our knowledge of Zr hydrides is useful for effectively controlling hydride embrittlement in fuel assembly. The aim of this review is to reorganize the mechanisms of hydride nucleation and growth behaviors, hydride reorientation under external stress, and hydride-induced embrittlement. We revisit important examples of progress of research in this field and emphasize the key future aspects of research on Zr hydrides.

## 1. Introduction

Zirconium (Zr) alloys exhibit excellent properties, such as high corrosion resistance, creep resistance, irradiation resistance, and low neutron-absorption cross-sections, and they have been widely used as nuclear-reactor fuel-cladding tubes and pressure vessels [1]. During service, α-Zr reacts with coolant water and generates hydrogen [2]. Zircaloy has a high affinity with hydrogen but has a very low hydrogen-solid solubility under ambient conditions [2]. The hydrogen uptake in Zircaloy precipitates as brittle hydrides, affecting the mechanical properties of Zircaloy matrices [3]. Neutron irradiation causes the expansion of nuclear fuel, which makes the cladding tube suffer a hoop stress of about 122–130 MPa [4]. During spent-fuel storage, Zr cladding also bears a tensile-hoop stress of about 150 MPa [5]. Under hoop tensile stress, the distribution of hydride usually alters from the original circumferential to the axial direction, which is known as hydride reorientation [6]. Reoriented hydride significantly reduces the mechanical properties of Zircaloys and reduces the service life of nuclear-fuel cladding and the safety storage of spent nuclear fuel [7,8]. Therefore, a comprehensive understanding of the nucleation, growth, reorientation, and embrittlement mechanism of hydrides is important for effectively controlling the detrimental effects of hydrides on Zr. Consequently, over several decades, both experiments and various simulation methods have been adopted to reveal hydrides’ behavior under different conditions and uncover the mechanism of hydride-induced embrittlement.

In this review, we briefly outline related research on Zr hydrides. Section 2 summarizes Zr hydrides’ nucleation and growth behavior. Section 3 reviews the measurement of the hydride-reorientation-threshold stress and the mechanism of hydride reorientation under external stress in Zr. Section 4 overviews the mechanical properties of Zr hydride and hydride-induced embrittlement. Finally, we briefly discuss some future research prospects.

## 2. Hydride Nucleation and Growth Behaviors

**Figure 1:**
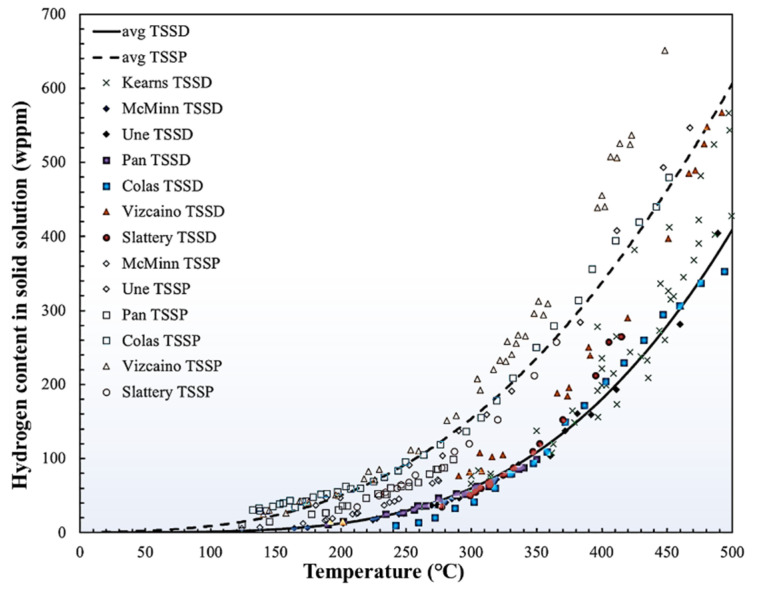
TSSD (solid line) and TSSP (dash line) in Zr and its alloys. Black lines represent average values of TSS based on previous studies [6]. Reprinted with permission from [6]; Copyright 2019 Elsevier.

Hydrogen is easy to gather and precipitates as hydride due to the low solid solubility of hydrogen in Zr [3]. The limit solid solubility of hydrogen in Zr has two categories: terminal solid solubility until the end of hydride dissolution (TSSD) during heating and terminal solid solubility at the start of hydride precipitation (TSSP) during cooling [9,10]. These TSSs can be measured by differential scanning calorimeter [11] and synchrotron radiation diffraction [12]. As shown in Figure 1, the average TSS in different Zr alloys is about 1 wppm at room temperature and rises to 100 wppm at service temperature, which is near 0.90 at.% [6]. Because of the extremely low solid solubility, hydrogen dissolved in Zircaloy tends to precipitate as hydride in a low temperature range (<100 °C). The volume mismatches between precipitated γ hydride or δ hydride and the matrix are about 12.3% and 17.2%, respectively [13]. The large dilatational misfit is sufficient to cause the emission of dislocations and dislocation loops in Zr matrices, accompanied by hydride precipitation [14]. Additionally, the movement of the hydride–matrix-phase interface also dissipates energy [15]. These two factors combine to delay hydride precipitation and cause TSSP to be significantly higher than TSSD (Figure 1). This phenomenon is named solubility hysteresis [12]. Both an experiment [16] and a simulation [17] suggested that hydride precipitation starts when the local hydrogen concentration reaches 5.9 at.%. Further details about hydride precipitation are discussed in Section 2.2.

### 2.1. Hydride Phases and Their Transition

**Figure 2:**
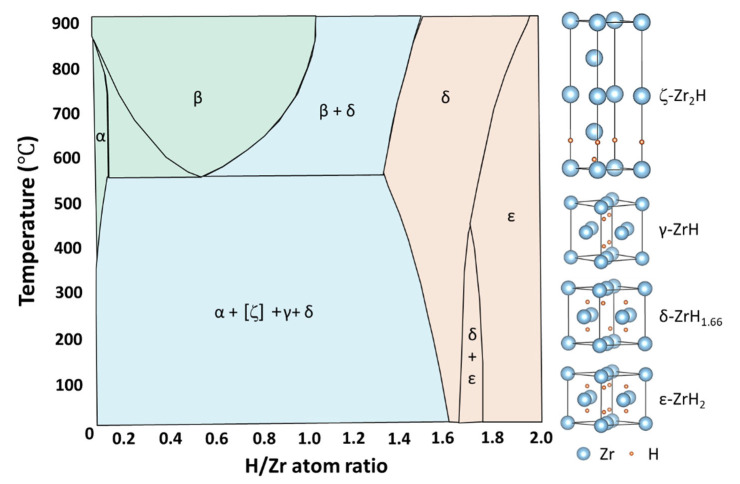
Zr-H phase diagram [3] and the unit cells of ζ-Zr_2_H, γ-ZrH, δ-ZrH_1.5_, and ε-ZrH_2_ [21,22].

Accompanied by hydride precipitation, different phases of hydride form. Despite multiple studies on this subject [18,19,20,21,22,23,24,25,26,27,28], there is still some controversy over the phase transition, stability, and formation preference of different hydride phases. There are four types of Zr hydride: ζ-Zr_2_H (Trigonal), γ-ZrH (FCT, c > a), δ-ZrH_1.5_ (FCC), and ε-ZrH_2_ (FCT, c < a) [18,19]. Figure 2 displays the Zr-H-phase diagram and the unit cell of the hydrides with the lowest energy state, according to a first-principles calculation [19,20,21,22]. Hydrogen atoms all tend to occupy the tetrahedral interstitial sites with the lowest binding energy rather than the octahedral sites in solid solutions or after forming hydrides [20,21]. The unit cell of ζ-Zr_2_H is composed of two HCP unit cells stacking along the [0001] direction and hydrogen occupying the two bottom layers of tetrahedral interstitial sites [18,21]. Hydrogen occupies different proportions of tetrahedral interstitial sites in FCC or FCT lattices to form γ-ZrH, δ-ZrH_1.5_, and ε-ZrH_2_ [22]. In particular, four hydrogen atoms inside the unit cell of γ-ZrH lie on one {110} plane. In a δ-ZrH_1.5_ unit cell, whole tetrahedral interstitial sites are occupied by hydrogen atoms, except for two hydrogen vacancies along the {111} direction.

According to the Zr-H phase diagram in Figure 2 [3], the dominant hydride phases below 500 °C are γ and δ hydrides. The δ hydrides generally form during slow cooling, while γ hydrides usually form in water-quenched samples [23,24]. Therefore, it is suggested that the δ hydride is an equilibrium phase and that the γ phase is a metastable phase. However, δ-ZrH_1.5_ slowly undergoes a eutectoid reaction with α-Zr to form γ-ZrH below 255 °C. After aging above 255 °C, γ-ZrH can also transform into α-Zr and δ-ZrH_1.5_ [24,25,26,27]. The revisable reaction invites a discussion on hydride stability [28]. Because of their similar Gibbs free energy, sensible enthalpy, and vibrational entropy, both γ-ZrH and δ-ZrH_1.5_ are energetically favorable precipitates at 0–327 °C [17]. Therefore, the transition from δ to γ below 255 °C is favored thermodynamically. The mechanism of the δ-to-γ phase transition is similar to the martensitic transformation of austenite-precipitated pearlite, relying on the slow diffusion of hydrogen atoms [25,27]. In addition, the preference of γ and δ hydrides varies under different conditions. Both γ-ZrH and δ-ZrH_1.5_ can nucleate through room-temperature hydrogen charging in pure Zr [29]. The δ-hydrides tend to form in Zircaloy under the influence of α-phase stabilizing elements (such as O, Fe, and Sn), whereas γ-hydrides are more likely to precipitate with the assistance of β-stabilizers (e.g., Nb) [30,31]. Under service conditions, δ hydrides with higher thermal stability and radiation resistance are more frequently detected and can also be used as moderators in nuclear reactors [32,33]. The stability and formation preference of γ and δ hydrides depend on various factors, such as the cooling rate, temperature, irradiation conditions, and alloying elements.

Phase transition occurs between hydride phases. With the increase in the knowledge on ζ hydrides, many studies focus on the transformation mechanism from ζ hydrides to γ/δ hydrides [18]. Bair et al., investigated the nucleation of δ hydrides on the basal plane and (10$\bar{1}$0) plane through phase filed modeling. It was found that ζ-Zr_2_H and γ-ZrH are potential precursors of δ hydrides [34]. Thuinet et al., proposed two precipitation sequences from ζ hydrides to γ hydrides, namely α → ζ-HCP → ζ-FCT → γ-FCT or α → ζ-HCP → γ-HCP → γ-FCT [35]. The generalized stacking fault energy (SFE) of the 1/3[10$\bar{1}$0]{0001} slip system for ζ-HCP is negative, whereas those of α-Zr and γ-HCP are positive [36,37]. Thus, the transformation from ζ-HCP into ζ-FCT phase is more likely to occur [38]; hence, the first path is preferred [36]. The δ hydride decomposes into ζ hydride during tensile loading [39], which has a fully coherent orientation relationship (OR) with the α matrix. Specially, the OR is described as (0002)_ζ_//(0002)_α_ and [2$\bar{1}\bar{1}$0]_ζ_//[2$\bar{1}\bar{1}$0]_α_. It can be concluded that ζ hydride is an important intermediate phase during δ hydride decomposition, and may also act as a nucleation precursor of δ hydride. However, because ζ-Zr_2_H is a thermodynamically metastable phase, the transition from ζ hydride to γ/δ hydride occurs immediately [17,18]. Capturing this transition during in situ experiments is still challenging.

In addition to the eutectoid reaction discussed above, δ hydrides can also transform into γ hydrides during tensile testing [40]. The γ phase is detected at the front of the δ hydride, implying that γ hydride is the precursor of δ hydride [41,42]. After long-term hydrogen charging at high temperatures, ε hydride is formed through the martensitic shear of δ hydrides [43,44,45,46]. The OR between δ and ε is fully coherent, such as <011>_δ_//<011>_ε_ and {111}_δ_//{111}_ε_ [45,46]. Zhu et al., suggested that γ transforms into δ, then δ transforms into ε with increasing hydrogen content through first-principles calculation [47]. The phase-transition sequence of γ → δ → ε was also confirmed in a hydrogen-charging experiment at room temperature [29].

### 2.2. Mechanism of Hydride Nucleation and Growth

**Figure 3:**
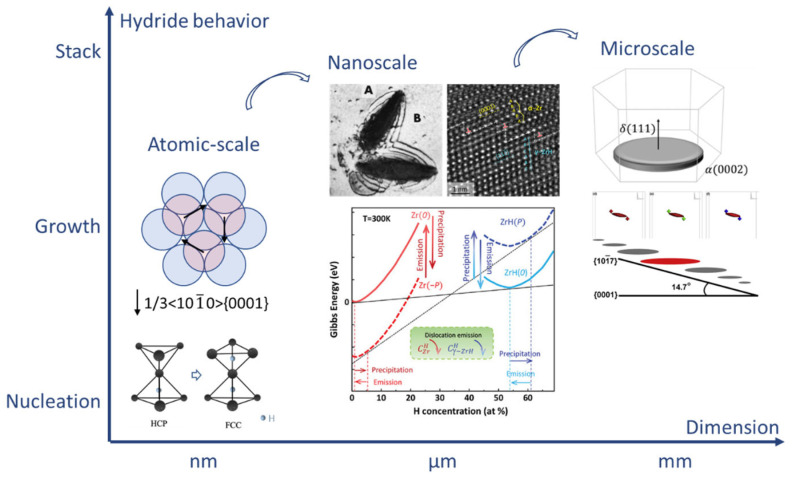
The mechanism of hydride behavior under different dimensions under stress-free conditions [6,14,52,56]. Reprinted with permission from [6]; Copyright 2019 Elsevier. Reprinted with permission from [14]; Copyright 1973 Elsevier. Reprinted with permission from [52]; Copyright 2019 Elsevier. Reprinted with permission from [56]; Copyright 2021 John Wiley and Sons.

Hydride maintains a special OR with α-Zr. The most common OR between hydride and α-Zr is (0001)_α_//(111)_Hydride_ and [11$\bar{2}$0]_α_//[110]_Hydride_, which is named first OR [6,13,23,36,38]. The habit plane of hydrides with first OR is the basal plane. Therefore, the distribution of hydrides can be tuned by controlling the texture of Zircaloy fuel cladding [48]. The hydride-orientation fraction is a key parameter in the evaluation of the quality of Zircaloy tubes [49]. Carpenter et al. [38] proposed a nucleation mechanism of hydride with first OR based on the movement of 1/3<10$\bar{1}$0>{0001} Shockley partial dislocation. In this way, a matrix with a HCP structure transforms into a FCT structure. Hydrogen subsequently diffuses to the tetrahedral interstitial sites of FCT structure, as illustrated in Figure 3. Furthermore, the SFE of the basal plane can be reduced from 200 mJ/m^2^ down to −60 mJ/m^2^, as hydrogen occupies 50% of the tetrahedral interstitial sites of the basal plane, enhancing the mobility of the Shockley dislocation [50]. Shinohara et al., confirmed this mechanism via an in situ transmission-electron microscope (TEM) experiment [51]. The increase in Shockley partial dislocations or the cross slip of perfect dislocations on the prismatic plane results in the formation of Shockley partial dislocation on alternate basal planes [51]. As these partial dislocations simultaneously slip, the habit plane of the mesoscale hydride transforms from {0001} to {10$\bar{1}$7}. The reason for this change in habit plane is further unveiled through phase-field modeling, as shown in Figure 3 [52]. The anisotropic interfacial energy and elastic energy together determine the plate-like spatial configuration of a single microscale δ hydride lying on the basal plane. The special distribution of elastic interaction energy around pre-existing δ-hydrides ensures that the upper-left and bottom-right corners of pre-existing hydrides are energetically favored nucleation locations for hydride variants. The original special stacking pattern shifts the entire growth direction of mesoscopic hydrides from the {0001} plane to the {10$\bar{1}$7} plane, with an interaction angle of 14.7°. Furthermore, the {111}<11$\bar{2}$> twin hydrides always have the first OR with a matrix [29]. Hydride grows gradually with the slip in the Shockley partial dislocation, causing the continuous accumulation of local strain at the hydride–matrix interface [29,53]. As a result, the gliding of Shockley partial dislocations with an opposite Burgers vector at the front of the interface is emitted, thereby producing twin hydride and effectively releasing local stress [29,53]. Therefore, under first OR, microscale hydrides constitute mesoscale hydrides through the alternate stacking of twin hydrides [29].

Dislocations play an important role in hydride nucleation [54]. The elastic strain energy and volume expansion generated by dilatational misfit stimulate the consecutive emission of dislocations and dislocation loops from the hydride–matrix interface. Local stress is reduced and hydride growth is further increased [51]. The density of the emitted dislocation is proportional to the square of the hydride length [42,55]. Liu et al., further pointed out the key role of dislocations in the regulation of hydride growth via an in situ experiment [56]. During hydride precipitation, compressive and shear back stresses accumulate inside hydride, while the tensile and shear stress gather in the α-Zr at the front of the interface. It was identified that local shear stress projected on slip planes is sufficient to activate all possible slip systems in α-Zr, eventually producing a butterfly dislocation configuration (see Figure 3). The effect of dislocation emissions on hydride growth is further analyzed by the Gibbs-free-energy curves in Figure 3. The accumulation of tensile stress at the hydride–matrix interface reduces the Gibbs free energy of Zr, which increases hydrogen solubility and stops hydride precipitation. Pouching-out dislocation decreases the tensile stress and increases the Gibbs free energy of Zr, which can reduce local equilibrium hydrogen solubility and reboot hydride precipitation. The growth rate of hydride sharply increases, and then gradually decays as the hydrogen source is depleted. The tensile stress and Gibbs energy at the interface need to be adjusted again via dislocation emission. Therefore, the precipitation of hydride follows an alternate sequence of dislocation emission and hydride growth, implying that dislocation-assisted hydride growth is a self-catalysis process.

**Figure 4:**
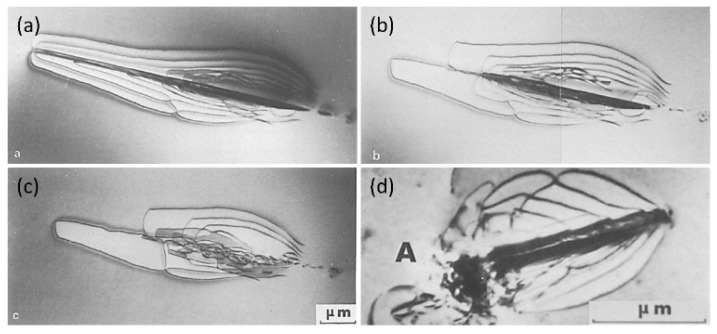
Precipitation–memory effect: (**a**) γ-ZrH at room temperature, (**b**) disappearance of γ-ZrH at 77 °C, (**c**) remaining dislocation structures at 117 °C, (**d**) reprecipitation of γ-ZrH after cooling from 142 °C to 42 °C [59]. Reprinted with permission from [59]; Copyright 1978 Elsevier.

In addition, it is generally believed that dislocations cause the precipitation–memory effect during hydride reprecipitation [57,58,59]. Below 400 °C, hydride gradually dissolves, and a small fraction of dislocations are recovered during heating [59], whereas most of the dislocation around hydride remains (Figure 4a–c) [59]. During cooling, the remaining <a> and <c> dislocations can act as heterogeneous nucleation sites for hydride, probably causing the precipitation–memory effect (Figure 4d) [42,59]. The precipitation–memory effect is also present in Zr-2.5Nb alloys [60,61,62]. After annealing at 350 °C, hydrides cross multiple phase interfaces and grain boundaries (GBs) and nucleate at unvarying sites. A variety of defect types can also be generated in the matrix during hydride precipitation [51]; therefore, the key defects leading to the precipitate–memory effect may need further investigation.

In addition to the first OR, there is a second OR between hydride and Zr matrix, (0001)_α_//(001)_Hydride_ and [11$\bar{2}$0]_α_//[110]_Hydride_ [29]. Weatherly et al., suggested that the habit plane of hydride with the second OR is the prismatic plane, and that hydride nucleation is related to the slip of the partial <a> dislocations on the prismatic plane [63]. Similarly, the hydrogen occupying 50% of the tetrahedral interstitial sites on the prismatic plane can decrease the SFE of partial <a> dislocations from 145 mJ/m^2^ to 67 mJ/m^2^ [50]. The formation mechanism of the second OR hydride was revealed based on a detailed analysis of high-resolution TEM images in FCC Zr [64] and Ti-H [65]. A slip of 1/6<11$\bar{2}$0>{10$\bar{1}$0} dislocation and atomic shuffle together assist the phase transition from HCP to FCT, with hydrogen migrating to the tetrahedral interstitial sites. For both types of OR, the misfit between the hydride and the matrix along different crystallographic directions is summarized in Table 1 [66]. The misfit is smallest along <11$\bar{2}$0>, which is the typical growth direction of hydrides.

### 2.3. Effects of Interface on Hydrides

The section above mainly discusses homogeneous intragranular hydride nucleation. However, heterogeneous hydride nucleation typically occurs at the interface, with a lower nucleation barrier. A typical interface in Zircaloy contains the phase interface, GB, and twin boundary (TB). In Zr-2.5Nb alloys, knowledge of the role of second phase (β) in the precipitation behavior of hydride is of great practical and scientific interest. In pure α-Zr, the diffusion coefficient of hydrogen can be calculated as [6]

(1) $$D_{H}=1.08\times 10^{-6}exp\left(-\frac{0.46\left(eV\right)}{\kappa_{B}T}\right)\left(m^{2}/s\right)$$

where $\kappa_{B}$ is Boltzmann’s constant and T is the temperature. When *T* = 277 °C, the diffusion coefficient is 6.58 × 10^−11^ m^2^/s, while the diffusion coefficient in Zr-2.5Nb alloys with layered α/β phase is equal to 9.63 × 10^−11^ m^2^/s [67]. The latter is 30% larger than the former. Furthermore, the solubility ratio of the hydrogen in β-Zr (Zr-20Nb) to that in α-Zr (Zr-0.6Nb) was found to range from 9 to 7 at 247–307 °C [68]. The large difference in hydrogen solubility and diffusion rate between the β phase and α phase creates a close link between the phase interface and hydride nucleation [69,70,71]. Krishna et al., found that hydrides preferred to nucleate at α/β-phase interfaces, obeying a Burgers OR, in a fully annealed Zr-2.5Nb alloy [72]. It was suggested that hydrides nucleated at the phase interface have two growth patterns [73]. One is extension along the interface, and the other is growth into α phase. These two types are defined as interphase hydrides and transgranular hydrides, respectively. Kim et al., reported a possible formation mechanism of interphase hydrides in Ti-6Al-4V [74,75]. Hydrogen atoms first dissolve in β phase, cause an increase in lattice spacing of about ~0.6%, and produce a local stress field near the interface. Local stress and low hydrogen concentrations at the α-hydride interface limit the initial hydride extension along the phase interface [74]. Interphase hydrides and the hydrogenated β phase encourage the initiation and rapid propagation of cracking, and eventually result in the early failure of Ti-6Al-4V alloys [74]. However, the mechanism underlying hydride precipitation in Zr-2.5Nb alloys still needs to be explored in more detail.

The nucleation preferences of intergranular and intragranular hydrides are relative. Early experiments focused on the effect of the stress state on hydride’s nucleation preference [76]. After a tensile strain of 4%, <11$\bar{2}$0>{10$\bar{1}$0} dislocation can emit more easily than <1$\bar{2}$13>{10$\bar{1}$1} dislocation, leading to compressive residual stress on the basal planes of grain interiors. Therefore, hydrides tend to precipitate at GBs and TBs. Conversely, after compression strain of 0.5% or 4%, {10$\bar{1}$2} tensile twins form to assist deformation along the <c> axis and relieve the compressive stress on the basal plane. Similarly, after annealing from the β region, intergranular thermal residual stress produces tensile stress on the basal plane [77]. These stress conditions favor the nucleation of intragranular hydride. Qin et al., proposed a formula to study the effect of GB structures on hydride nucleation [78]

(2) $$\frac{J_{GB,b}^{*}}{J_{intra}^{*}}=\left(\frac{3\rho \Lambda }{d}\right)\exp\left[-\frac{\Delta G_{b}^{*}-2r_{0}^{2}\sigma_{aa}-\Delta G_{intra}^{*}}{\kappa T}\right]$$

where $J^{*}$ is the nucleation rate. When $J_{GB,b}^{*}/J_{intra}^{*}$ > 1, intergranular hydrides dominate; otherwise, intragranular hydrides are preferred. The ρ is the GB width. The Λ is the probability that the GB is parallel to the basal plane. These two parameters can be set as constant in macroscale statistics. The d is the grain size. The volume ratio of the GB decreases as the grain size increases, leading to a decrease of $J_{GB,b}^{*}/J_{intra}^{*}$ [53]. The $\Delta G_{b}^{*}$ and $\Delta G_{intra}^{*}$ are the critical nucleation energies for intergranular and intragranular hydrides, respectively. The $\sigma_{aa}$ is the GB energy. For pure α-Zr, c-axis misorientation is more representative of the GB character than GB misorientation [29]. With c-axis misorientation of less than 15°, the GB energy is low. Thus, collective shear can take place on the basal planes of adjacent grains, causing intergranular-hydride nucleation [53]. When c-axis misorientation is greater than 80°, high GB energy is conducive to hydride nucleation [78]. For c-axis misorientation near 60°, the interaction angle corresponds to the angle between the basal plane and the {10$\bar{1}$1} secondary habit plane of the hydride, increasing the nucleation of intergranular hydride [79]. In general, hydrides tend to grow at GB with c-axis misorientation less than 15°, equal to 60°, and greater than 80°.

**Figure 5:**
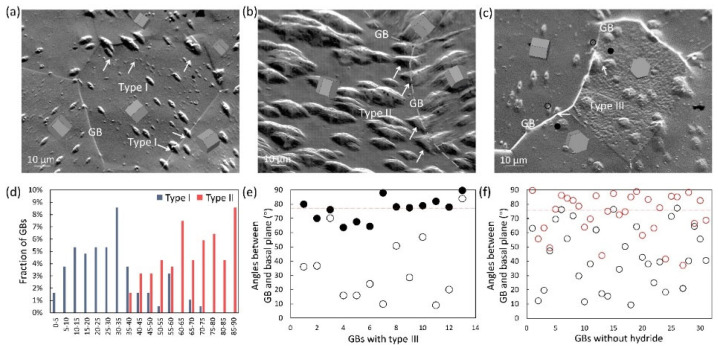
Dependence of intergranular hydride on GB structure [29]. (**a**–**c**) SEM images showing three types of intergranular hydride bump; (**d**) distribution of type I and type II hydride bumps vs. c-axis misorientation; (**e**) distribution of type III hydride bumps vs. GB-plane–basal-plane angle; (**f**) characteristics of GBs without hydride. Reprinted with permission from [29]; Copyright 2021 Elsevier.

In addition, it is also important to clarify the classification of intergranular hydride and the influence of GB structures on intergranular hydride types [53,78,80]. Recently, Jia et al., investigated the morphology and distribution of intergranular hydrides and categorized them into three types (Figure 5a–c) [29]. Type I hydride spans the GB and keeps a similar growth direction. Type II hydride involves two different growth orientations and appears to intersect at the GB. Type III hydride either grows only on one side of the GB or lies solely within the GB. Type I and Type II hydride depends on the c-axis misorientation of GBs (Figure 5d). Hydrides can cross the GB with a c-axis misorientation lower than 40° and maintain the same growth direction. This explains why mesoscale hydrides on the circumferential–radial plane of strongly textured Zircaloy tubes can cross several grains, displaying a linear morphology over 100 μm in length and almost perfect alignment [6]. The GBs with Type III hydride (Figure 5e) and GBs without hydride nucleation (Figure 5f) all have GB planes that are nearly perpendicular to the basal planes of adjacent grains. The GBs with this characteristic can effectively inhibit hydride nucleation. These observations guide the application of GB engineering and control over hydrogen damage in Zircaloy materials.

As a semi-coherent interface, TB is a special GB. There are few studies on hydride nucleation on TBs, and some contradictory conclusions are suggested. Wang et al., found that hydrides easily nucleated on the TB of a <10$\bar{1}$1>{10$\bar{1}$2} (T1) twin in a fully recrystallized Zr-4 [53]. However, Arunachalam et al., pointed out that no hydride nucleation was observed on the TBs in an annealed Zr-2 [81]. In deformed Zircaloy, Perovic [76] and Kim [82] reported the nucleation preference of hydrides on TB. The residual dislocation around TB probably encourages the preferential nucleation of hydrides [82]. Therefore, twins formed under different fabrication processes may influence the hydride nucleation and should be analyzed further.

### 2.4. Effect of Irradiation Defect on Hydride Formation

During service in nuclear reactors, Zircaloy is exposed to high-energy neutron irradiation. Numerous point defects and various dislocation loops are produced, causing typical irradiation effects, such as irradiation growth and irradiation hardening [83]. Therefore, it is necessary to explore the relationship between hydrogen, hydrides, and irradiation defects.

**Figure 6:**
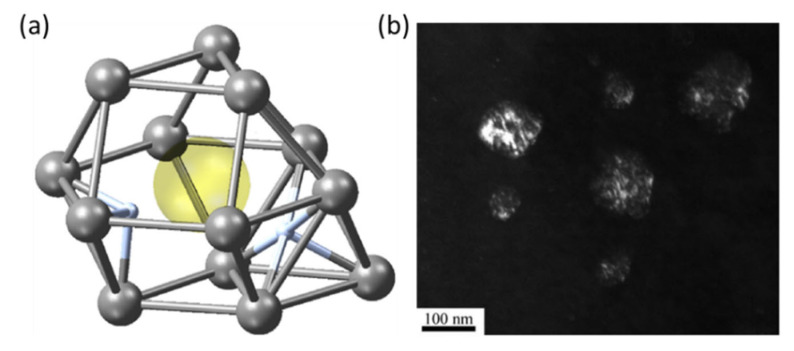
(**a**) Hydrogen atoms (blue) around a vacancy (yellow) of Zr lattice in three-fold (left) and five-fold (right) coordinates [21]; (**b**) fark-field image of circle-like hydride [89]. Reprinted with permission from [21]; Copyright 2020 Elsevier. Reprinted with permission from [89]; Copyright 2019 Elsevier.

Irradiation-induced defects affect the TSS of hydrogen. The <a> and <c> dislocation loops delay hydride precipitation by trapping hydrogen atoms. After 4 h of post-irradiation annealing at 600 °C, δ-hydride appears, accompanied by the disappearance of dislocation loops [84]. Furthermore, numerous vacancies are created during irradiation [83]. A self-vacancy in α-Zr has a total of six five-fold sites and eight three-fold sites for hydrogen, as shown in Figure 6a [21]. It was reported that the binding energy of each hydrogen atom was near 0.20 eV for nine hydrogen atoms and decreased, as appropriate, to 0.0 eV for the five remaining hydrogen sites. Therefore, a vacancy can stably trap nine hydrogen atoms [85]. Consequently, irradiation-induced defects can effectively increase the solubility of hydrogen [86]. In turn, hydrogen atoms also affect the evolution of irradiation defects. For example, hydrogen atoms encourage the transition from two-dimensional vacancy platelets to <c> dislocation loops through their effect on the surface energy of crystal planes in HCP Zr [87], and increase the stability of <c> dislocation loops by decreasing the *I_1_*-type basal SFE [88].

Irradiation defects significantly change hydride microstructures. Hydrides tend to nucleate and precipitate around the short dislocations and dislocation loops produced during irradiation. Hydride volume decrease and hydride density increase by several orders of magnitude [48,89]. Li et al., found that <a> and <c> dislocation loops, as hydrogen traps, can induce the formation of circular-like hydride (Figure 6b) [89]. The OR between circular hydride and the matrix also changes from first OR to (01$\bar{1}\bar{1}$)_α_//(200)_δ_ and [01$\bar{1}$2]_α_//[011]_δ_.

Due to the retarding effect of irradiation defects on hydride deformation, the hardness of δ and ε hydrides increases to 1.2 and 1.7 times after 5 dpa irradiation, respectively [90]. During deformation, the hardened hydride phase and hydride–matrix interface can serve as channels for rapid crack propagation [5]. After irradiation, the ultimate tensile strength of a sample with 250 ppm hydrogen reduced from 600 MPa to 350 MPa, and the plastic hoop strain decreased from 40% to 12%. Therefore, irradiated hydride further degrades the mechanical properties of Zr matrix.

## 3. Hydride Reorientation under External Stress

**Figure 7:**
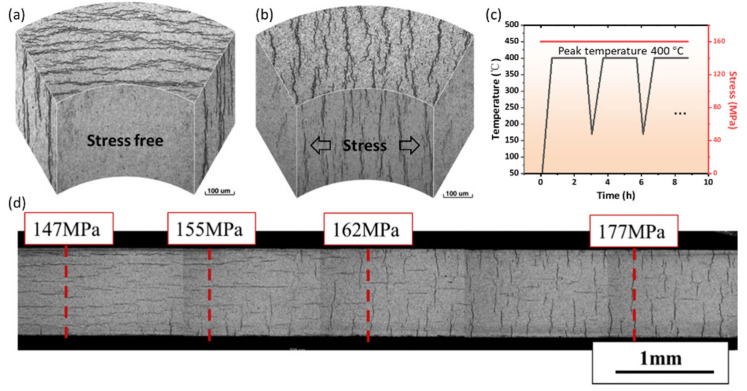
(**a**) Circumferential hydride; (**b**) radial hydride formed after 8 cycles of treatment at 400 °C (peak temperature) and under 160 MPa of tensile stress; (**c**) heat-treatment schedule of the sample [91]; (**d**) distribution of hydride in the cross-section of a tapered uniaxial tension sample after 2 cycles of heat treatment [93]. Reprinted with permission from [91]; Copyright 2008 Elsevier. Reprinted with permission from [93]; Copyright 2016 Elsevier.

Hydrides tend to redistribute in a direction perpendicular to the tensile stress when a Zr specimen is cooled down under stress from the temperature at which hydrides are dissolved. This is called hydride reorientation [91]. Generally, for Zircaloy tubes, hydrides formed under zero stress are called circumferential hydrides, and reoriented hydrides are named radial hydrides, as shown in Figure 7a,b. Radial hydride returns to circumferential hydride after annealing without stress, implying that tensile stress is the key factor causing hydride reorientation [58,62,92].

### 3.1. Threshold Stress for Hydride Reorientation

The threshold stress for hydride reorientation is defined as the applied tensile stress at which hydride reorientation starts, which is generally lower than the yield strength of the Zr matrix [94,95]. Hydride-reorientation experiments are usually conducted using the thermal cycling treatment, as plotted in Figure 7c [91]. The tubes’ internal and external pressures were regulated by a constant-differential-pressure-control system to maintain constant values of hoop tensile stresses during cycling [91]. At the end of reorientation treatment, all hydrogen should be precipitated as hydrides [94]. Various factors, such as the grain microstructure, texture, specific fabrication history, peak temperature (Figure 7c), hydrogen content, and matrix-stress state influence the threshold stress of hydride reorientation [91,93,96,97,98,99,100,101,102,103,104,105]. Here, we mainly discuss the last three factors. First, hydrogen diffusion along the radial orientation becomes faster as the heat-treatment peak temperature increases, leading to a lower threshold stress. After 2 h of solution annealing with 450 °C as the peak temperature, more than 90% of dissolved hydrides are reoriented under 160 MPa of hoop stress [91], whereas only 10% of hydrides reorient after annealing at a peak temperature of 300 °C. This effect is caused by the residual surrounding defects of undissolved circumferential hydride. These defects can hinder radial-hydrogen diffusion and capture dissolved hydrogen to induce the memory effect of circumferential hydride re-precipitation [91,105]. Second, the effect of hydrogen content on threshold stress is very complex [96,106]. When the hydrogen content is lower than the TSSD at peak temperatures, all the hydrogen atoms solute in the matrix and can readily diffuse along the radial direction to assist in reorientation. Therefore, threshold stress decreases with increases in hydrogen content. When the hydrogen content is higher than the TSSD, the hindering effect and the re-precipitation–memory effect together induce an increase in the reorientation stress with further increases in the hydrogen concentration. Overall, when the hydrogen content is near that of the TSSD, the threshold stress for hydride reorientation is lowest. The typical hydrogen concentration in a spent fuel cladding ranges from 300 to 600 wppm [8], which is higher than the TSSD at the service temperature of 400 °C during dry storage (Figure 1). Thus, the threshold stress generally increases with increasing hydrogen concentrations. Third, the stress state of the matrix significantly affects the threshold stress of hydride reorientation [93]. Under uniaxial tension, the threshold stress for hydride reorientation is about 155 MPa (Figure 7d). Under plane-strain conditions, the threshold stress drops to 110 MPa. Under near-equibiaxial tension, the threshold stress is only 75 MPa. The stress state of the matrix during spent-fuel storage is close to plane-strain conditions; hence, hydride reorientation easily occurs and become more detrimental [93].

In order to measure the degree of reorientation, various parameters have been proposed, as listed in Table 2 [96]. A complete metric should indicate the precise description of the reorientation morphology and the susceptibility of cladding to radial-hydride-induced embrittlement. In addition to examining the hydride microstructure, the degree of reorientation can also be measured by X-ray bt comparing the full width at half maximum (FWHM) of circumferential and radial hydrides [107,108].

### 3.2. Mechanism of Hydride Reorientation

**Figure 8:**
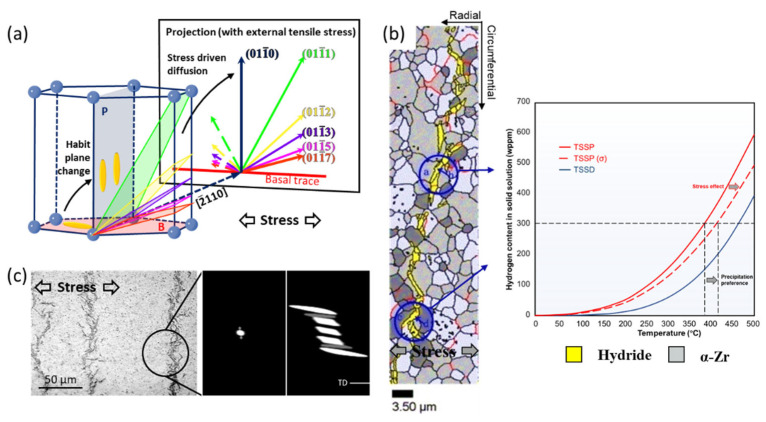
Mechanisms of hydride reorientation. (**a**) Stress drives hydride reorientation at microscale [66]; (**b**) change in nucleation sites when TSSP decreases under large tensile stress [114,115]; (**c**) hydride stacking perpendicular to the tensile stress [6,52]. Reprinted with permission from [66]; Copyright 2022 Elsevier. Reprinted with permission from [115]; Copyright 2006 Elsevier. Reprinted with permission from [6]; Copyright 2019 Elsevier. Reprinted with permission from [52]; Copyright 2019 Elsevier.

The stress state of the Zr sample affects the nucleation and stacking of hydrides, and also induces hydride reorientation. At the microscale, the habit plane of reoriented hydride gradually shifts from the {0001} plane to the {10$\bar{1}$i} (i = 0–7) planes under different degrees of tensile stress, as shown in Figure 8a [66,109,110,111]. However, how tensile stress induces such a transition in the habit plane remains intriguing. One explanation [66] is that a higher tensile stress on the {10$\bar{1}$i} planes than on the {0001} plane reduces the volume mismatch between the hydride and the matrix, accelerating hydrogen diffusion and increasing hydride precipitation on the {10$\bar{1}$i} planes. Phase-field modeling revealed that applied external stress alters the direction of hydrogen diffusion by modifying the stress field around hydride [112]. Furthermore, some research attributes the transformation of habit planes to the change in OR between the hydride and the matrix [109,110,113]. This means that the nucleation sites of hydrides shift from the {0001} to the {10$\bar{1}$i} plane, especially at the crack tip [113].

At the mesoscale, two explanations have been proposed for hydride reorientation. First, tensile stress can significantly lower the elastic strain energy at the hydride–matrix interphase. Solubility hysteresis is subsequently reduced via the variation of the TSSP (Figure 8b). Grain interiors in which basal plane was perpendicular to the tensile direction and GBs with the GB plane facing in the tensile direction are the regions that bore the largest tensile stress in a sample (Figure 8b) [114,115]. In these regions, hydride precipitates earliest during cooling. This proposal highlights that stress changes hydride’s preferred precipitation sites rather than its crystallographic characteristics, leading to hydride reorientation [116]. Second, alterations in the hydride stacking pattern contribute to hydride reorientation. The stacking and combination of hydrides clustered along the direction perpendicular to that of the tensile stress can reduce the threshold stress for hydride reorientation to ~100 MPa [117]. This value is much lower than the threshold stress of 9 GPa of a single hydride and is near the experimental values in previous studies [91,93]. This large difference suggests that hydride stacking has a significant effect on the threshold stress for hydride reorientation. As shown in Figure 8c, microscale hydrides generally stack along the direction perpendicular to the tensile stress to form a mesoscale-reoriented hydride [6]. The special hydride stacking direction is attributed to the preferred nucleation sites of microscopic hydrides, which change from the upper-left and the bottom-right corners of pre-existing hydrides (Figure 3) to the middle position under tensile stress (Figure 8c) [52]. Under 350 MPa of applied tensile stress in the circumferential direction, the hydride-stacking orientation exhibits an interaction angle with the basal plane of approximately 75°. These mechanisms indicate that hydride reorientation is a complex process, which is affected by the preferences of hydrogen diffusion, the deflection of the habit plane, the variation in the stress state around the hydride, the transitions of the nucleation site, and the adjustment of the stacking sequence.

## 4. Hydride-Induced Embrittlement

In this section, we present an overview of the mechanism of hydride-induced embrittlement in Zr. The proportion of the hydride-induced deterioration of the matrix’s mechanical properties is highly dependent on the distribution and orientation of the hydrides [5]. We focus on four parts, as follows: the mechanical properties of hydrides, delayed hydride cracking (DHC), local hydride embrittlement, and reorientated hydride embrittlement.

### 4.1. Mechanical Properties of Hydrides

**Figure 9:**
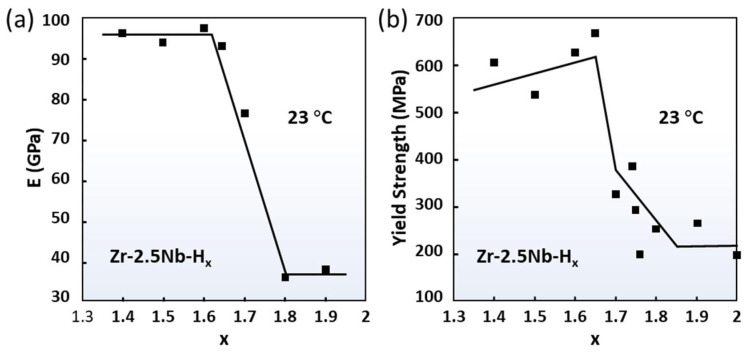
The Young’s modulus (**a**) and yield strength (**b**) of ZrH_x_ [118]. Reprinted with permission from [118]; Copyright 2005 Elsevier.

Nanoindentation is performed to measure the mechanical properties of hydrides. Figure 9 plots the Young’s modulus and yield strength of ZrHx (x = 1.3–2.0) [118]. When x is between 1.3–1.6, the hardness of hydride remains about twice that of pure Zr. When x is between 1.6–1.8, located at the stage of phase transition from δ-ZrH_1.5_ to ε-ZrH_2_, the Young’s modulus and yield strength of the hydride rapidly drop. When x is greater than 1.8, all the phases are ε hydride. The hardness of ε hydride drops to about 70% of that of α-Zr [119]. Notably, the fracture toughness of ε hydride is only 0.63 MPa∙m^1/2^ [119,120]. The fracture toughness of δ hydride is 1 MPa∙m^1/2^ at room temperature and rises to 3 MPa∙m^1/2^ at 300 °C [121]. These values are much lower than those of Zr alloys (>40 MPa∙m^1/2^), indicating that hydrides are very brittle [119]. To reveal the brittle nature of hydrides, the surface energy $\gamma_{S}$ and unstable stacking fault energy $\gamma_{US}$ of the slip plane of α-Zr and hydride were calculated [37]. The $\gamma_{S}$ of the {111} plane in δ-hydride was 25% smaller than the $\gamma_{S}$ of the prismatic plane in Zr, implying the easy generation of a fracture surface in hydride. In contrast, the $\gamma_{US}$ is larger than the $\gamma_{US}$ of prismatic plane in Zr, which means that dislocation motion is very difficult. Therefore, hydride has greater hardness and much lower toughness than α-Zr. Observations made from experimental studies indicated that precipitated hydride can obstruct the gliding of dislocations, which causes a decrease in the creep rate and induces hydride embrittlement [122,123,124].

### 4.2. Delayed Hydride Cracking

**Figure 10:**
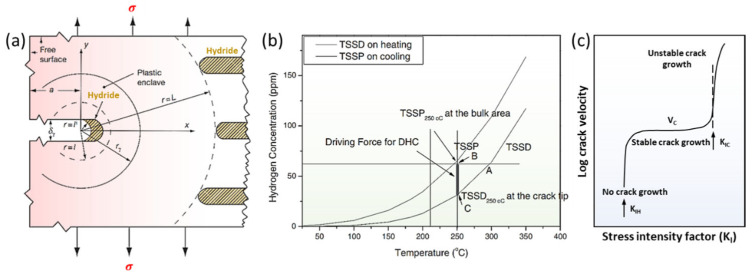
(**a**) Diffusion first model; (**b**) precipitation first model; (**c**) DHC velocity vs. applied stress-intensity factor [126,127]. Reprinted with permission from [126]; Copyright 2009 Elsevier. Reprinted with permission from [127]; Copyright 2008 Elsevier.

The mechanism of delayed hydride cracking (DHC) was first proposed by Dutton and Puls, as illustrated in Figure 10a [125]. Puls reiterated and further developed the model [126]. In Zircaloy, the diffusion equation of hydrogen can be described as

(3) $$J_{H}=-\frac{C_{H}D_{H}}{\Omega_{Zr}RT}\nabla \mu_{H}^{D}$$

where$\;C_{H}$ is the atomic fraction of hydrogen in α-Zr, $D_{H}$ is the diffusion coefficient of hydrogen in α-Zr, $\Omega_{Zr}$ is the atomic volume of Zr in α-Zr, and $\mu_{H}^{D}$ can be calculated using

(4) $$\mu_{H}^{D}\left(r,p\right)=\mu_{H}^{0}+RT\ln c_{H}^{D}\left(r,p\right)-p\left(r\right)\cdot {\bar{V}}_{H}$$

In Equation (4), $\mu_{H}^{D}\left(r,p\right)$ represents the chemical potential under arbitrary hydrostatic stress, $p\left(r\right)$. The $c_{H}^{D}\left(r,p\right)$ is the concentration of the diffusible hydrogen. The R and T have their usual meaning. The ${\bar{V}}_{H}$ is the molar volumes of the hydrogen solution in α-Zr and $-p\left(r\right)\cdot {\bar{V}}_{H}$ is the interaction energy during the hydrogen solution. Based on Equation (4), the chemical potential at zero stress is

(5) $$\mu_{H}^{D}\left(r,0\right)=\mu_{H}^{0}+RT\ln c_{H}^{D}\left(r,0\right)$$

To reach equilibrium between the region at zero stress and at arbitrary stress, the chemical potential given in Equations (4) and (5) should be equal, which means that the hydrogen concentration in the stressed part continuously increases according to

(6) $$c_{H}^{D}\left(r,p\right)=c_{H}^{D}\left(r,0\right)\cdot exp\left(p\left(r\right)\cdot {\bar{V}}_{H}/RT\right)$$

Based on Equations (4)–(6), the chemical potential decreases under tensile stress, driving hydrogen diffusion to stretched region. When hydrogen in bulk precipitates as hydrides (Figure 10a), a similar derivation process is built [126]. It was mathematically proven that the gradients in chemical potential rather than the concentration gradients are the fundamental drivers of hydrogen diffusion. Therefore, driven by chemical potential, dissolved hydrogen from hydride at bulk diffuses from the interior matrix to the crack tip (Figure 10a). Hydrogen continuously accumulates and re-precipitates as reoriented hydride at the crack tip, resulting in a continuous decrease in the fracture toughness of the matrix. As local stress is sufficient to fracture the reoriented hydride, cracks propagate along the hydride until encountering the Zr bulk, at which point they are blunted. The hydrogen in the interior matrix continues to diffuse towards the blunt crack tip until fracture conditions are re-established. The fracture of matrix displays an intermittent process of “hydrogen diffusion–hydride precipitation–crack propagation”. Thus, it is called DHC [125]. Cann et al., demonstrated that hydrides always accumulate and grow at the crack tip, along the direction perpendicular to that of the tensile stress [113]. After the hydride reaches a certain length at the crack tip, the crack further expands. The DHC is a process involving the repeated precipitation and fracture of hydrides at the crack tip at the microscale [113].

The debate about the driving force in hydrogen diffusion (the first step in DHC) lasted for a long period. The above explanation regarding the chemical potential as the driving force is called the diffusion first model (DFM) [125,126]. Kim et al., doubted that the agglomeration of hydrogen concentrations at the crack tip is insufficient to reach the local TSSP [127,128,129,130]. Based on the concentration differences derived from TSS data under different stress conditions, Kim claimed that another mechanism drove the first step in DHC [128]. The qualitative explanation is that solubility hysteresis decreases with the increase in tensile stress, as explained in Section 3.2 (Figure 8b). Thus, the spontaneous nucleation of hydride is prompted by the creation of plastic deformation at the crack tip at the start of each DHC crack-propagation step. The prioritized nucleation creates a hydrogen-concentration gradient between the crack tip and the bulk, which is the driving force in hydrogen diffusion (Figure 10b). This model is called the precipitation first model (PFM). These two mechanisms therefore provide opposite explanations for the driving force in hydrogen diffusion. However, more theoretical understanding has been developed based on the diffusion first model [131,132], and many experimental observations can be quantitatively described by the diffusion first model [133,134,135]. Hence, the diffusion first model is more widely accepted and comprehensive in explaining DHC.

Current DHC experiments mainly focus on four points. The first is to describe the crack-propagation process by characterizing the fracture surface [136]. The second is to observe the precipitation and fracture behavior of hydrides on the cross-section during in situ DHC testing [137]. The third is to maintain a constant load to measure the crack-growth velocity V_c_ during stable crack-growth stage (Figure 10c) [138]. The fourth is to measure the stress-intensity factor K_IH_ in the initiation of DHC using up-down-loading methods (Figure 10c) [139].

### 4.3. Local Hydride Embrittlement

**Figure 11:**
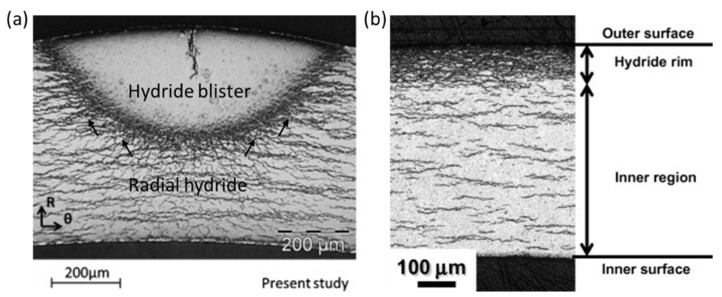
(**a**) Hydride blister [140]; (**b**) hydride rim [141]. Reprinted with permission from [140]; Copyright 2014 Elsevier. Reprinted with permission from [141]; Copyright 2011 Elsevier.

Driven by stress or temperature gradients, hydrogen is locally accumulated in areas with greater tensile stress or lower temperatures. As a result, hydride blisters [140] (Figure 11a) and hydride rims [141] (Figure 11b) are formed underneath the oxide layer. A water-cooled point setup is often employed to develop local blisters in Zircaloy materials [140,142]. Hydride blisters usually contain, at most, about 80% δ-ZrH_1.5_ and part of α-Zr, and the δ-ZrH_1.5_ and Zr meet the first OR [26,143,144]. Due to the large volume misfit at the blister–matrix interface, a hoop tensile stress of about 320 ± 90 MPa is produced in the surrounding matrix [145]. The hoop stress is high enough to induce radial-hydride precipitation at the interface [140,142] (Figure 11a). Cracks initiating from the blister interior can easily propagate along the surrounding hydrides [146].

The effect of local hydride precipitation on the mechanical properties of the matrix has attracted attention [147]. Mechanical tests, such as the Expansion Due to Compression test, and tensile tests were conducted on samples containing localized hydride and demonstrated that hydride blisters and hydride rims have no deformation capacity, even at 480 °C [148,149,150,151,152]. Notably, the reduction in the strength and plasticity of the matrix is proportional to the depth of the bister/rim. The matrix finally fractures in a brittle manner when the ratio of the blister/rim depth to the tube thickness reaches above 30% [148,149,150,151,152]. Hydride rims with the same thickness are more harmful than hydride blisters [149]. Undoubtedly, localized hydride can drastically reduce the mechanical integrity of the matrix and become an initiation area for DHC during service [147]. In 1983, the failure of the Zircaloy-2 pressure tubes in the CANDU reactor was due to the cold spots formed upon the contact between the pressure tube and the calandria tube; consequently, hydrogen accumulated and precipitated as an array of hydride blisters [147]. Cracks initiated from the hydride blisters, which further broke the tube wall and extended along the axial length of the tube to a length of 2 m [147]. Therefore, the measurement of K_IH_ and the checking of the operating temperature are important issues for controlling the initiation stage of DHC in samples with blisters/rims [153].

### 4.4. Hydride Reorientation Embrittlement

**Figure 12:**
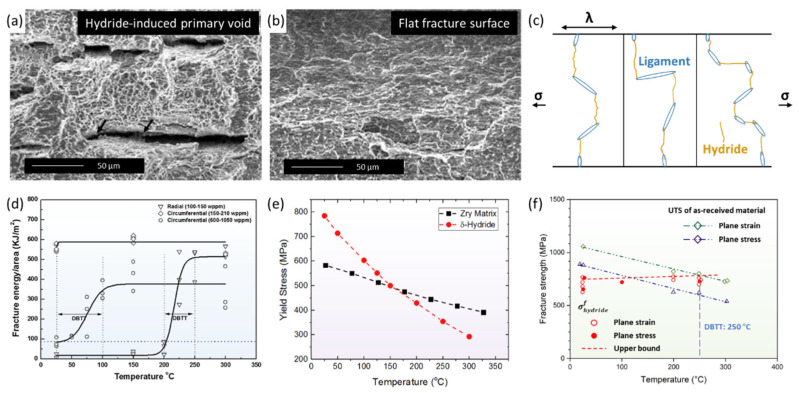
Fracture surface and ductile-to-brittle transition (DBT) mechanism of sample with radial or circumferential hydrides. The fracture surface of Zr-4 containing (**a**) circumferential hydride and (**b**) radial hydride at room-temperature four-point bending test [155]; (**c**) the connectivity of hydride network [6]; (**d**) DBT temperature of Zr-4 with radial and circumferential hydrides [157]; (**e**) yield stress of Zr-4 and circumferential δ hydride vs. temperature [165]; (**f**) fracture strength of radial δ hydride vs. temperature [166]. Reprinted with permission from [155]; Copyright 2012 Elsevier. Reprinted with permission from [6]; Copyright 2019 Elsevier. Reprinted with permission from [157]; Copyright 2015 Elsevier. Reprinted with permission from [165]; Copyright 2020 Elsevier. Reprinted with permission from [166]; Copyright 2013 Elsevier.

As mentioned above, circumferential hydrides can seriously damage the mechanical properties of the matrix only at high hydrogen concentrations or with localized precipitation [147]. In particular, the fractographs of Zircaloy materials containing circumferential hydrides and radial hydrides are quite different (Figure 12a,b) [154,155,156,157,158,159,160]. The fracture surface displayed in Figure 12a has a quasi-cleavage fracture of micropores, cavities, and facets [155]. Micropores formed in the circumferential hydride, after which elongated voids and cracks gradually began to appear. The overall deformation is a damage-accumulation ductile fracture. Only with considerable circumferential hydrides can cracking penetrate the entire cross-section [156]. The fracture surface shown in Figure 12b is relatively flat, with numerous secondary cracks under 200 °C, suggesting a typical cleavage fracture [155]. Radial hydride provides propagation channels for primary and secondary cracks, encouraging the failure of samples due to brittleness [157]. Qin and Motta et al. [6,111,161,162] proposed a mechanism in which radial-hydride-induced embrittlement is linked to the impact of hydride-network connectivity on crack propagation (Figure 12c). Hydride connectivity is defined as the remaining ligament after the easiest path through the cladding within a length λ (Figure 12c) [6], which can also be measured by the radial-hydride-continuity factor, as listed in Table 2 [96,157,163]. Undoubtedly, radial hydrides result in lower connectivity compared to circumferential hydrides of the same size. Moreover, the rupture critical strain of radial hydrides ranges from 1.5% to 10% for a length/width ratio between 10 and 70, while that for circumferential hydrides increases to 1.5%–30% [164]. This suggests that radial hydrides are more likely to rupture than circumferential hydrides. As a result, when radial hydrides’ connectivity drops to 30–43%, cracks can easy appear inside them, pass through jointed hydride clusters, and propagate in the remaining ligament, leading to the brittle fracturing of the matrix [6,102,163].

In addition, the ductile-to-brittle transition temperatures (DBTTs) of samples containing circumferential hydrides or radial hydrides have different ranges, as shown in Figure 12d [102,157]. When a circumferential hydride’s concentration reaches 600–1050 wppm, the sample undergoes DBT in a wide range of 0–100 °C. The DBT mechanism is related to the variation in the yield stress of the matrix and δ hydride with the increase in temperature, as displayed in Figure 12e [165]. Before DBT, the yield strength of the hydride is higher than that of the matrix; therefore, the hydride phase contributes to 10–12% of the total strain energy at 20 °C. The total deformation is dominated by hydride in a brittle manner. As the temperature increases, the yield strength of the hydride rapidly drops, whereas that of the matrix slightly decreases. The different temperature responses cause the strain–energy ratio of the hydride to drop to less than 1% at 200 °C. The Zircaloy matrix generally dominates the deformation of the sample in a ductile manner; therefore, DBT slowly occurs [165]. The DBTTs of samples containing 100–150-wppm radial hydrides arises within the range of 50 °C; however, the mechanism is still unclear. Kubo et al., evaluated the fracture strength of radial δ-hydrides at temperatures between 25 °C and 250 °C [166]. These hybrids demonstrated a very slight temperature dependency and remained at about 710 MPa, as shown in Figure 12f. Around 250 °C (DBTT), the ultimate tensile strength of the matrix decreased to less than 710 MPa. The Zircaloy matrix undergoes ductile fracture prior to the fracturing of radial hydrides. This observation suggests that the increase in matrix deformation is the main factor inducing DBT [12]. Furthermore, another explanation suggests that hydride can undergo plastic deformation at high temperatures, which dominates the ductility recovery of the matrix and the occurrence of DBT [155,157,167]. Despite these theoretical analyses, however, more experiment results are needed to support the proposals based on them.

## 5. Conclusions and Prospects

The hydrides in α-Zr and Zircaloy were studied in past decades and remain essential issues today. The review provides a brief overview of the mechanism of hydride precipitation and hydride-induced embrittlement. Generally, hydrogen has low solubility in α-Zr and tends to precipitate as hydrides in a sequence of ζ-Zr_2_H → γ-ZrH → δ-ZrH_1.5_ → ε-ZrH_2_. The EELS technique is important in the identification of evidence of ζ-Zr_2_H phase. The self-catalyzing growth of hydrides with two types of OR is highly dependent on the emission of dislocations. Hydrides tend to nucleate at GBs with c-axis misorientation of less than 15°, equal to 60°, and greater than 80°. Furthermore, GBs with planes perpendicular to the adjacent basal plane can inhibit hydride nucleation. Hydrides have two growth patterns in duplex-phase Zr-2.5Nb alloys, interphase hydrides, and transgranular hydrides. Hydrides prefer to nucleate at TBs with dislocation. Irradiation defects can increase hydrogen solubility, induce circular hydride formation, and harden hydride to further aggravate hydride-induced embrittlement. The tensile stress on special crystalline planes initiates hydride reorientation. The question of how multi-machining factors and service conditions affect threshold stress is well-researched. However, the reorientation mechanism is perplexing and complicated, as it involves various adjustments, including hydrogen diffusion, the hydride habit plane, the nucleation site, and the stacking sequence. Different models of embrittlement, such as the diffusion first model, precipitation first model, and hydride-network connectivity, were proposed based on the high hardness and extremely low fracture toughness of hydrides.

As discussed above, diverse efforts have been made to understand hydrides in Zr and Zircaloy materials. However, there some open issues still need to be resolved. Below, we describe four representative examples.

(1) Clarifying the mechanism of hydride reorientation

The threshold stress for hydride reorientation can be precisely measured [91,93,96], but the specific effect of tensile stress on hydride reorientation is still unclear. It is necessary to perform in situ observations and explore the link between microscale- and the mesoscale-reorientated hydrides. Furthermore, the influence of interface and irradiation defects on hydride reorientation requires further investigation [5,98,110,168].

(2) Revealing the effect of reoriented hydrides on DBTs in Zr

Reoriented hydrides narrow the DBTT range of the matrix [102,157], but a reliable mechanistic explanation and direct experimental evidences are still lacking. Conducting in situ or post-detailed observations on Zircaloy materials around DBTTs could help to clarify these issues.

(3) Discovering the effect of alloying elements on Zr hydride

Matrices influence hydride behaviors. In Zr-2, ZIRLO, Zr-2.5Nb, and other alloys, the hydride characteristics, the reorientation degree, and the DBTT are different under similar test conditions [6,102]. A clear understanding of the underlying mechanism would assist in the development of a management system for the spent-fuel storage of different alloys [102].

(4) Strengthening the atomic-scale simulations of Zr hydride

In Zr-H simulations, phase-field modeling is conducted to describe the formation and stacking of hydrides [52,112,117]. The thermodynamics and crystal structures of hydrides can be calculated by using first principles [17,22]. Hydride models have been established by using the finite element method [2,169,170,171]. However, molecular-dynamics simulations are insufficient [36], and the potential of Zr-H systems needs to be further developed [172,173].

## Figures and Tables

**Table 1:** The misfit between the hydride and Zr matrix with two different ORs [66]. Reprinted with permission from [66]; Copyright 2022 Elsevier.

OR	[uvtw]_α_	[uvw]_hydride_	Misfit between γ and α (%)	Misfit between δ and α (%)
{0001}//{111} <11$\bar{2}$0>//<110>	[0001]	[111]	5.69	7.25
	[11$\bar{2}$0]	[110]	0.54	4.60
	[1$\bar{1}$00]	[112]	5.64	4.58
{0001}//{001} <11$\bar{2}$0>//<110>	[0001]	[001]	−3.48	−7.09
	[11$\bar{2}$0]	[110]	0.54	4.60
	[1$\bar{1}$00]	[110]	16.14	20.82

**Table 2:** Various parameters proposed to quantify the degree of hydride reorientation [96]. Reprinted with permission from [96]; Copyright 2018 Elsevier.

Parameter	Definition	Calculation Formula	Variable
F_n_(40)	Fraction of the number of radial hydrides	$\frac{\sum N_{40}}{\sum N}$	$N_{40}$ Number of hydrides in the radial direction ±40°
			N Number of all hydrides
F_1_(45)	Fraction of the length of radial hydrides	$\frac{\sum L_{45}}{\sum L}$	𝐿45Length of hydrides in the radial direction ±45°
			L Length of all hydrides
RHF	Radial-hydride fraction	$\frac{\sum_{i}L_{i}f_{i}}{\sum_{i}L_{i}}$	$f_{i}$ Weighting factor; value for hydride in the radial direction between 0–35° is 1, value for the direction between 35° and 50° is 0.5, and value for the direction between 50–90° is 0.
RHCF	Radial-hydride continuity factor	$\frac{L_{R}}{h_{m}}$	𝐿𝑅Maximum length of the continuous radial hydride
			$h_{m}$ Cladding-wall thickness

## Data Availability

Not applicable.
